# Anti-Proliferative Activity of Poloxamer Cobalt Ferrite Nanoparticles against Human Prostate Cancer (DU-145) Cells: In-Vitro Study

**DOI:** 10.1049/2024/8929168

**Published:** 2024-03-20

**Authors:** Nazanin Oroskhani, Seyed Mohammad Amini, Sakine Shirvalilou, Mehdi Khodaie, Seyed Rabi Mahdavi

**Affiliations:** ^1^Department of Medical Physics, School of Medicine, Iran University of Medical Sciences, Tehran, Iran; ^2^Radiation Biology Research Center, Iran University of Medical Sciences, Tehran, Iran; ^3^Finetech in Medicine Research Center, Iran University of Medical Sciences, Tehran, Iran; ^4^Materials Science and Engineering, K. N. Toosi University of Technology, Tehran, Iran

## Abstract

Prostate cancer is the second most frequent type of cancer death in men. This study refers to the novel hyperthermia application of poloxamer-coated cobalt ferrite as a new approach for thermal eradication of DU-145 human prostate cancerous cells under a radio frequency magnetic field (RF-MF). The hydrothermal method was applied for the synthesis of cobalt ferrite nanoparticles. Then, the structure, size, and morphology of nanoparticle were characterized. The cytotoxicity of the synthesized nanoparticles and RF-MF exposure on DU-145 prostate cancer cells was investigated separately or in combination with colony formation methods and MTT [3-(4,5-Dimethylthiazol-2-yl)-2,5-Diphenyltetrazolium Bromide] assay. Transmission electron microscopy (TEM) confirmed the spherical morphology of nanoparticles with a size of 5.5 ± 2.6 nm. The temperature of cells treated with nanoparticles under RF-MF reached 42.73 ± 0.2°C after 15 min. RF-MF treatment or nanoparticles have not affected cell viability significantly. However, the combination of them eradicated 53% ± 4% of cancerous cells. In-vitro hyperthermia was performed on human prostate cancer cells (DU-145) with cobalt ferrite nanoparticles at specific concentrations that demonstrated a decrease in survival fraction based on colony formation assay compared to cells that were treated alone with nanoparticles or with RF-MF.

## 1. Introduction

Eradicating cancer cells without harming healthy cells has been a primary aim in chemotherapy and radiation therapy. The side effect of chemotherapy and radiation treatment on normal tissue, multidrug resistance and radiation resistance, problems in biological issues to concentrate chemotherapy and radiation treatment of some tumors, etc. have reduced the treatment efficiency of prostate cancer [1]. It has been demonstrated that hyperthermia could enhance the chemo or radiation treatment of PCa [2]. Historically, heat has been transferred to cancerous cells using external applicators, either in the form of ultrasound, electric field, or microwaves [3]. However, energy generated by these methods creates unwanted hotspots in normal tissue because it is difficult to focus the energy on the target area [4]. As a result, there is a continuing need for new hyperthermia application techniques with better heating properties [5]. Therefore, new approaches must be developed to supplement or replace existing procedures of treatment. Nowadays, there are various modern techniques based on nanoparticles that show fewer side effects, hyperthermia induced by an external magnetic field (magnetic hyperthermia (MH)) is an example of them [5, 6]. MH uses magnetic nanoparticles (MNPs) to generate local heat in the presence of the alternating magnetic field. This technique could increase the tumor region temperature up to 42–45°C at a certain time, which leads to tumor cell eradication [7]. Also, another advantage of MNP biomedical applications is the ability to accumulate MNPs within the tumor through a magnetic targeting strategy for cancer therapy. Another outstanding feature of MH is that it includes frequencies that are not harmful to the body (typically 50–1,000 kHz) and heats the points where the nanoparticles are present [8]. MNPs are attractive for cancer hyperthermia, due to their special magnetic properties [9]. In this study, the cobalt ferrite nanostructure was selected, because of higher magnetic crystal anisotropy. Also, cobalt ferrite nanoparticles with spinel structure have good magnetic permeability, fair coercive force (more than 50KOe), medium saturation magnetization (80 emu.g^−1^), good mechanical hardness, high magneto-optical effect, high curie temperature (*T*_C_), good electromagnetic performance, and excellent stability of physical and chemical properties [9, 10]. We know that at a special temperature called the curie temperature, ferromagnetic materials transform into paramagnets. In this way, ferromagnetic materials lose their strong magnetic properties. Therefore, it is possible to improve hyperthermia. By designing and manufacturing MNPs with *T*_C_ just above 45°C, i.e., 318°K, the heating stops automatically upon reaching the curie temperature. Therefore, nanoparticles act not only as mediators but also as keys to controlling temperature in vivo [11]. Therefore, these nanoparticles are good candidates for MH due to their good magnetic moment, moderate biocompatibility, and great specific absorption rate (SAR) for killing cancer cells [12]. A study by Islam et al. [13] on cobalt ferrite nanoparticles showed that Néel relaxation and Brownian relaxation are the heating mechanism of cobalt ferrite nanoparticles, which is smaller than 18 nm. The heating mechanism of bigger nanoparticles is hysteresis loss. Synthesis of MNPs with suitable SAR that has a biocompatible surface coating can be a suitable platform for the development of MH. In this study, we used a cobalt ferrite MNP with poloxamer coating to investigate the hyperthermia index. For this purpose, we investigated the synergetic effect of MNPs and radio frequency magnetic field (RF-MF) on DU-145 cells. Therefore, the colony formation and MTT assay were used to assess combined methods.

## 2. Materials and Method

Iron (III) nitrate nonahydrate and cobalt (II) hexahydrate were the sources of metal ions. Ammonia (25%) was used to adjust the pH. All items were prepared by Merck. RPMI culture medium, fetal bovine serum (FBS; Gibco, Grand Island, NY), penicillin–streptomycin (Sigma–Aldrich, St Louis, MO), and EDTA 0.25% Trypsin (EDTA; Sigma–Aldrich) were prepared. Poloxamer polymer was prepared from Sigma–Aldrich (USA).

### 2.1. Synthesis and Characterizations of Nanoparticles

#### 2.1.1. Synthesis of MNPs

The preparation of MNPs was performed by a hydrothermal method in the previous study [14]. Briefly, iron (III) nitrate nonahydrate and cobalt (II) hexahydrate were applied as a metal precursor. Ammonia (25%) was utilized for pH adjustment. All reactions were conducted in deionized water (DI water). The molar ratio (Fe/Co = 2.1) of iron and cobalt salt was mixed in DI water. Then, citric acid (CA) was added to this solution in a molar ratio equal to the oxidizer (fuel/metal cation molar ratio = 1.1). The pH of the reaction was set to 10 by adding ammonia. The obtained solution was transferred to a teflon-lined stainless steel reactor encompassed by heating elements and heated for 20 hr at 160°C temperature. After the system had been cooled, the obtained nanoparticles were washed with a series of centrifugation and decantation with DI water and dried in a 60°C overnight. Then, the dried nanoparticles were dispersed in DI water containing 0.1 M poloxamer 407 solutions with 1 hr constant and vigorous shaking.

#### 2.1.2. Characterization of Nanoparticles

For assessing the size and morphology of nanoparticles, a drop of nanoparticle solution was placed on the carbon grid. The grid was dried and imaged by TEM (Zeiss LEO 906,100 kV). In addition, DLS was used to obtain the hydrodynamic diameter of the nanoparticle (NANO-flex Particle Sizer Germany). The zeta potential (Zeta-check, Microtrac, Germany) determines the surface charge of nanoparticles that could affect the various biological responses to the nanoparticles. Also, the zeta potential increases and the repulsion between the charged particles increases and makes the particles more stable [15]. X-ray diffraction (XRD) Rigaku—Ultima IV X-ray diffractometer with CuK_*α*_ source (*k* = 1.541 A, 40 kV, and 40 mA) was used for XRD analysis.

### 2.3. Cell Lines and Monolayer Culture

The human prostate cancer cell line DU-145 was obtained from the Pasteur Institute (Tehran, Iran). RPMI-1640 culture medium was selected for DU-145 cells, which contained 10% FBS and 1% 1% penicillin–streptomycin. A value of 250 × 10^4^ cells was cultured monolayer in a T-25 flask. The flask was kept in an incubator (37°C, 5% CO_2_). One milliliter of PBS was used to wash the cells and then detached with 1 ml of EDTA 0.25% Trypsin.

### 2.4. Cytotoxicity of Cobalt Ferrite Nanostructure

The 96-well microplate and third cell passage were used for this assay. It should be 10,000 cells/well. After a day in the incubator, the cells were impregnated with the determined concentrations (0−1−2−4−8−16−32−64−128−256, and 512 *μ*g/ml) and maintained in the incubator for 24 hr. Then, cold PBS was used to wash the cells. Hundred microliter of MTT dye was added to each well, and the plate was maintained in the incubator for 4 hr (with a concentration of 5 mg MTT dye in 1 ml PBS). After removing the MTT-containing medium and replacing it with dimethyl sulfoxide (100 *μ*l per well), the necessary conditions for the plate to continue the test were provided. The conditions were room temperature and darkness for 15 min on the shaker. Then, the optical density was read by an ELISA reader (570 nm, Garni Medical Eng, Iran).

### 2.5. Heating Measurement and Hyperthermia Treatment

The temperature increase of DU-145 cells that have been treated with cobalt ferrite nanoparticles under RF-MF waves was investigated. The cells were cultured in two groups for 24 hr, one of the groups was incubated with nanoparticles of concentration 512 *μ*g/ml for 1 day. Then, the group containing nanoparticles was washed with PBS and replaced with a fresh medium. The second group did not contain nanoparticles. Both groups, with and without NPs, were irradiated to RF-MF (LAB A, HT-1000W, 400 KHz, NATSYCO). The system applied a coil, and magnetic field strengths were applied with 9.23 kA/m and field frequencies of 400 kHz. The current of the device is sinusoidal, and the continuous magnetic field created is also sinusoidal. Each exposure cycle was 3 min, and the power was 1,000 W. Temperature changes in the groups were measured with a digital thermometer every 3 min. MH depends on the value so-called SAR, which represents the efficacy of the MNPs' energy absorption from the exposed magnetic field. SAR can be calculated by this formula:

(1) $$\begin{array}{c} \text{SAR}=C\left(\frac{\text{dT}}{\text{dt}}\right)\left(\frac{m_{\text{s}}}{m_{\text{m}}}\right). \end{array}$$

*C*: specific heat capacity of suspension, (dT/dt): initial slope of the temperature curve, *m*_s_: the total mass of the suspension, and *m*_m_: the mass of magnetic material content in suspension. According to our previous study, the maximum SAR is equal to 10.63 W/g [14]. Finally, after RF-MF, the percentage of cell survival was evaluated for different groups and they were prepared for colony formation assay (CFA).

### 2.6. Colony Formation Assay

After treating the mentioned groups on the cells, the cells were grown in 60 mm petri dishes containing 5 ml of complete medium. After 10 days, RPMI was removed. Formaldehyde (2%) was applied to fix the cells for 20 min. Crystal violet dye (0.5%) was used for 60 min to stain the colonies. Finally, colonies were counted. Equations (1) and (2) demonstrated plating efficiency (PE) and surviving fraction (SF), respectively:

(2) $$\begin{array}{c} \text{PE }\left(\%\right)=\frac{\text{Number of colonies counted}}{\text{Number of cells seeded}}\times 100, \end{array}$$

(3) $$\begin{array}{c} \text{SF}=\frac{\text{Colonies counted}}{\text{Cells seeded}\times \frac{\text{PE}}{100}}. \end{array}$$

### 2.7. Statistical Analysis

All experiments were repeated thrice. Statistical analysis of the results was done with GraphPad Prism six software (GraphPad Software, Inc., San Diego, CA). ANOVA and student *t*-test were used to check the significance of the results. A value of *p* < 0.05 was considered statistically significant between various treatment and control group.

## 3. Results

### 3.1. Characterization of Cobalt Ferrite Nanostructure

**Figure 1:**
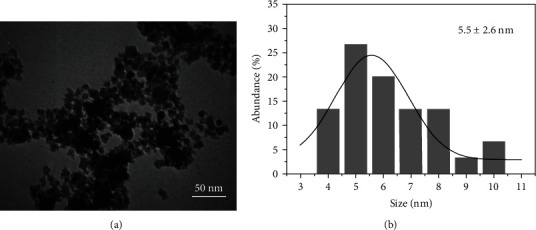
TEM image of cobalt ferrite nanoparticles (a). The size distribution of the nanoparticles is 5.5 ± 2.6 nm (b).

**Figure 2:**
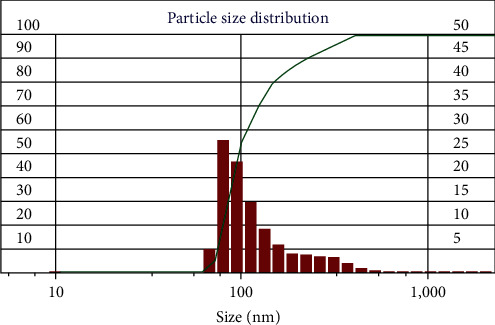
The hydrodynamic analysis of cobalt ferrite nanostructure.

Figure 1(a) shows a TEM micrograph of the cobalt ferrite nanostructure. TEM micrographs show that the size of nanoparticles is homogeneous and also all nanoparticles are almost spherical. Digital micrograph software was used to calculate the size distribution of nanoparticles. The average diameter of synthesized NPs is 5.5 ± 2.6 Figure 2(b). Moreover, Figure 2 illustrates the hydrodynamic diameter of the NPs, representing that the NPs' size was 97.6 nm. Zeta potential showed NPs were −16 mV, indicating good stability of NPs. Nanoparticles must be able to maintain their dispersed phase as a suspension or easily re-dispersed if precipitated, which is the concept of nanoparticle stability. The stability of particles can be achieved in two ways, electrostatic and steric. Particles with a zeta potential greater than ±30 mV are usually considered electrostatically stable [16, 17].

**Figure 3:**
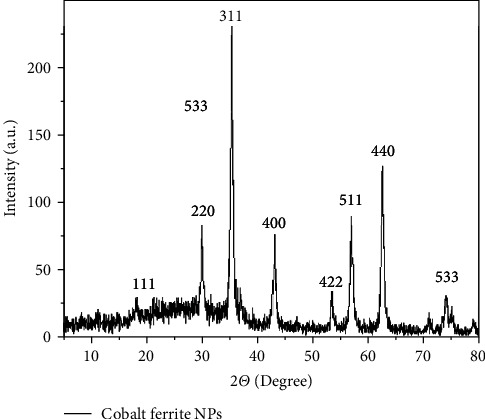
XRD pattern of synthesized cobalt ferrite nanoparticles.

The XRD pattern of the cobalt ferrite NPs was examined against 96−591−0064 and 96−900−6921 JCPDS cards (Figure 3). The nanoparticle crystal system was identified as an inverse spinel cubic structure.

### 3.2. Cytotoxicity Assessment of Cobalt Ferrite

**Figure 4:**
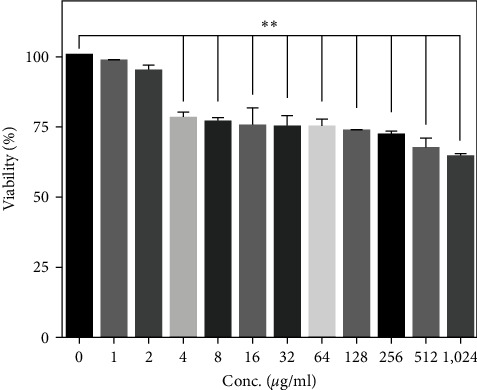
Cell survival percentage of DU-145 cells treated with different concentration of cobalt ferrite (Student *t*-test was applied for analysis of the data, compared to the control group, asterisks represent significance in  ^*∗∗*^*p* < 0.01).

The MTT results depicted in Figure 4 represent that no cytotoxic effect was observed for up to 2 *μ*g/ml of NPs. According to Figure 4, as the concentration increased, the percentage of cell viability decreased. However, more than half of the cells remained alive even in the highest selected concentration (512 *μ*g/ml). So, the concentration of 512 *μ*g/mL with 66.91% cell survival was selected for other tests.

### 3.3. Heating Response of Cobalt Ferrite under RF-MF

**Figure 5:**
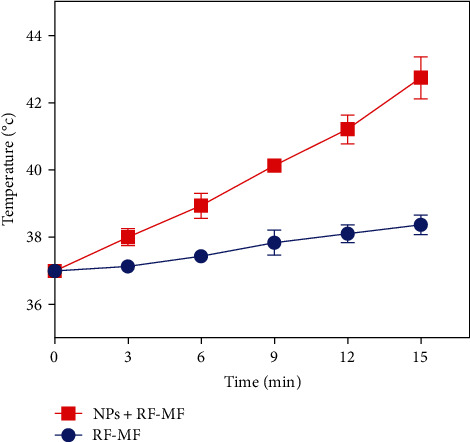
Temperature curve of DU-145 cells induced by RF-MF with and without of nanoparticles.

Figure 5 represents the heating response cells with and without nanoparticles according to the duration of RF-MF exposure. It was observed that the NP group reached the temperature that is necessary for hyperthermia treatment (42.7°C) after 15 min of RF-MF exposure, while cells without nanoparticles experienced a temperature of 34.2°C. The average temperature of the cells treated with cobalt ferrite nanoparticles increased faster under the induction of RF-MF. So, the initial slope of the temperature change graph increased from 0.08°C/min in the absence of nanoparticles to 0.38°C/min in the presence of nanoparticles, which made it possible to reach the wanted temperature in a shorter time.

### 3.4. MH Assessment

**Figure 6:**
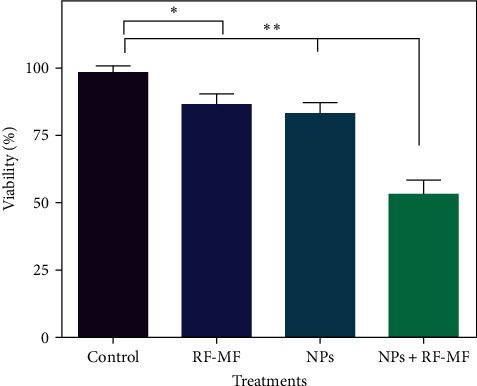
Survival percentage graph of DU-145 cells after 15 min of RF-MF in the presence and absence of cobalt ferrite nanoparticles. (Student *t*-test was applied for analysis of the data, asterisks represent significance in  ^*∗*^*p* < 0.05 and  ^*∗∗*^*p* < 0.01). NP, nanoparticle; RF-MF, radio frequency magnetic field.

The viability of cells was measured to evaluate the hyperthermia effectiveness of RF-MF and cobalt ferrite nanostructure.  Figure 6 shows the viability of DU-145 cells treated with MNPs either alone or with RF-MF for 15 min. As we expected, a significant difference was obtained for the group of nanoparticles alone and the group of nanoparticles with RF-MF exposure. Also, the survival percentage of DU-145 cells for the NPs+RF-MF treatment group was impressive, and its average was 53%.

### 3.5. Effects of MH on CFA

**Figure 7:**
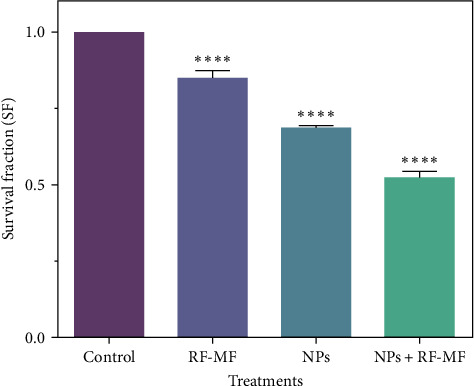
Colony survival ratio (CFA) of DU-145 cells after different treatments. The significance of the groups has been compared with the control group (one-way ANOVA  ^*∗∗∗∗*^*p* < 0.01).

CFA is for assessing survival division and self-renewal. The CFA of cells (with and without NPs) diminished within the presence of RF-MF; therefore, fewer colonies were found in exposure to RF-MF (15 min) and nanoparticles (512 *μ*g/ml). CFA analysis (Figure 7) showed that the RF-MF treatment group is moderately significant, and nanoparticles alone could not kill all the DU-145 cells. However, the combination of RF-MF exposure causes more cell death and, as a result, decreases the survival rate.

## 4. Discussion

After the synthesis of nanoparticles, characterizations were performed. TEM micrographs demonstrated the spherical shape of cobalt ferrite nanoparticles with a diameter of 5.5 ± 2.6 nm. However, DLS measured the hydrodynamic size distribution to be 98 nm. It is clear in the provided TEM micrograph that the small clusters of the nanoparticles are flocculated tougher. However, the boundaries of each nanoparticle are recognizable. This flocculation of the nanoparticles could be a result of the magnetic field of the TEM apparatus. In general, based on the TEM micrograph (Figure 1) and the high value of hydrodynamic diameter, it could be concluded that the nanoparticles are prone to aggregate. It has been reported that few aggregates could lead to a very high value of the hydrodynamic value of nanoparticles [18]. The cobalt ferrite nanostructures are aggregated as a result of the magnetism properties of nanoparticles [19, 20]. A study by Jabir et al. [21] on Fe_3_O_4_ nanoparticles coated with polyethylene glycol (PEG) with a size of 9–12 nm, which are almost spherical like our nanoparticles, demonstrated that coating with PEG causes a decrease in the size and better dispersion of nanoparticles. Also, it has been found that uncoated Fe_3_O_4_ nanoparticles have aggregated, but they were well dispersed following coating with PEG [21]. Zeta potential analysis showed a negative value (−16 mV), which indicates its low electrochemical stability. The electrostatic stability was achieved for NPs with ±30 mV surface charge [22]. However, steric stabilization of the nanoparticles could be achieved by coating the nanoparticles with various coating agents including natural compounds [23, 24], polymers [25, 26], silica [15, 16], and various surfactants [17, 22]. Here, the poloxamer coating was applied to achieve the steric stabilization of nanoparticles. PEOx-PPOy-PEOx is the general expression for block copolymers that are generally referred to as poloxamers, where *x* and *y* represent the total average number of PEO and PPO repeat units, respectively. This arrangement gives rise to amphiphilic copolymers [27]. As a result of peg presentation in the block copolymer, steric stabilization was achieved by poloxamer coating on the surface of the nanoparticles [28]. When the nanoparticles have been dispersed in water-based solutions, it will be precipitated after a few hours. So, the synthesized cobalt ferrite nanoparticles are stabilized with a poloxamer coating to increase the steric stability of the nanoparticles. Poloxamer is an emulsifying agent that has been applied for solubilizing various nanoparticles such as magnetic particles [29].

Our MTT results showed that after the incubation of DU-145 cells with nanoparticles for 24 hr, the survival of cells for concentrations of 1 and 2 *μ*g/ml was not significantly different from the control group. With increasing concentration, this difference became statistically significant in the rest of the investigated concentrations. However, the amount of cell death is negligible even in the highest investigated concentration (512 *μ*g/ml). Peeples et al. [30] investigated the difference between the toxicity of bare iron oxide and bare cobalt ferrite nanoparticles with stabilized nanoparticles with two coatings of 3-aminopropyl triethoxy silane and CA. They achieved the extent of the cell damage caused by the concentration of nanoparticles (5, 25, 50 *μ*g/ml) by performing an MTT assay on lung epithelial cells. Iron oxide nanoparticles with both coatings are less toxic compared to uncoated iron oxide. In the case of cobalt ferrite nanoparticles, the coating of these nanoparticles significantly decreased the toxicity, even at the highest investigated concentration [30]. Another report has shown that cobalt ferrite nanoparticles coated with poly (maleic anhydride-alt-1-octadecene) were mildly toxic ranging from 80% to 100% in the various concentration range (1.56, 3.15, 6.25, 12, and 25 *μ*g/ml) on sarcoma 180 cancer cells [31]. Ali et al. [32] studied the toxicity of gold nanoparticles with concentrations of 2, 6, 12.5, 25, 50, and 100 *μ*g /ml on breast cancer cells. They also reached about 80% cell death at a concentration of 100 *μ*g/ml [32].

The toxicity of chitosan (CS)-coated cobalt ferrite nanoparticles was investigated by Nam et al. [33] The biocompatibility of the CS-coated on HepG2 and MCF7 cell lines with 48 hr incubation time in different concentrations (12.5, 25, 50, 75, 100, 150, and 300 *μ*g/ml) was determined with sulforhodamine B assay. They reported cell survival decreased with increasing concentration of nanoparticles [33]. Similar to the reviewed studies, poloxamer coating in the present study reduced the toxicity of the particles so that most of the cells survived in the highest concentrations examined.

The magnetic heating mechanisms for magnetic particles are related to two relaxation mechanisms, Neel and Brownian. In the nanocrystal, the rotation of the magnetic moment around the crystal axis creates the relaxation of Neel. The conversion of magnetic field energy into thermal energy under an alternating magnetic field is caused by the rotation of the magnetic moment. The rotation and friction of particles in the solution under the induction of an alternating magnetic field produce an energy called Brownian relaxation [34]. Because the size of our nanoparticles is below the critical size, both mechanisms are involved.

The use of radio frequency magnetic to heat cells and kill them by hyperthermia with nanoparticles has been reported [35]. Examining the heating of cobalt ferrite nanoparticles by RF-MF in Mobaraki's study showed that these nanoparticles reached a temperature of 45°C in 15 min (1,000 W, 300 KHz). They showed that the simultaneous combination of cancer cells with cobalt ferrite nanoparticles and magnetic field exposure significantly improves the cytotoxic effect and also increases Bax gene expression and decreases HSP70 and Bcl_2_ genes [36]. In the current study, the temperature increase of the DU-145 cells under RF-MF is significantly higher in the presence of poloxamer-coated cobalt ferrite nanostructure.

Hoque et al. [37] investigated the survival percentage of cells with spinel ferrite nano ensembles (MnFe_2_O_4_, CoFe_2_O_4_, and Fe_3_O_4_) in gliosarcoma cancer cells under RF-MF (400 kHz and 76 mT) up to 60 min. They observed more cell death with longer RF-MF application time [37]. In the study of KAHIL using cobalt ferrite nanoparticles, they investigated the survival of carcinoma cells under hyperthermia. Also, they reached 10% cell death with 60 min of exposure [38]. However, the nanoparticles could reach the hyperthermia range in 15 min. In 2019, Hadi et al. [39] studied the effect of combined cancer treatment including radiotherapy and RF-MF (13.56 MHz; 100 W; 15 min) and iron oxide nanoparticle mediators as thermal sensitizers. They observed that with the concentration of 20 *μ*g/ml of these nanoparticles, the temperature can be reached at 43°C. The obtained results showed that NPs and RF-MF alone were not effective, while together they had significant effects on cell survival and induction of programmed death [39]. Fantechi et al. [40] evaluated the survival of mouse B16 melanoma cells under a magnetic field (12.4 kA/m, 183 kHz) using the CoFe_2_O_4_ MNPs loaded in human ferritin protein (CoFe_2_O_4_–HFt). The results of this study and examination of cell survival with colony assay, MTT, and trypan blue tests showed 70% cell death in 60 min of incubation time and 45% death in 30 min of incubation time [40]. Medina et al. investigated the TNBC cell lines through microwave irradiation-based hyperthermia (2.45 GHz) of the cobalt ferrite nanoparticles causing more cell death compared to the case where there is no microwave radiation [41].

We measured the temperature of the DU-145 cells during RF-MF exposure to investigate the heating response in the presence of cobalt ferrite nanoparticles. When cells are exposed to RF-MF, their average temperature increases over time, and the average temperature of cells treated with cobalt ferrite nanoparticles increases much faster than untreated cells. The colonization ability of cells after different treatments was evaluated by CFA. The results indicated that the use of hyperthermia with MNPs significantly decreased the survival fraction in the CFA. We showed that cobalt ferrite nanoparticles can sensitize DU-145 cells to RF-MF exposure, which is MH.

## 5. Conclusion

A novel poloxamer-coated cobalt ferrite nanoparticle generated a considerable temperature rise in oscillating RF-MF exposure. The diameter of synthesized nanoparticles was 5.5 ± 2.6 nm, and the surface charge of the particles was −16 mV based on Gaussian analysis of TEM micrographs and zeta potential measurement, respectively. Hyperthermia studies demonstrated that the separate treatment of poloxamer-coated cobalt ferrite or RF-MF hyperthermia had no significant effect. However, together, they had an impressive effect on the elimination of prostate cancerous cells. It can be concluded that poloxamer-coated cobalt ferrite under RF-MF eradicates the cancerous cells effectively.

## Data Availability

All data are provided in manuscript.
